# *Helicobacter pylori* infection associates with fecal microbiota composition and diversity

**DOI:** 10.1038/s41598-019-56631-4

**Published:** 2019-12-27

**Authors:** Fabian Frost, Tim Kacprowski, Malte Rühlemann, Corinna Bang, Andre Franke, Kathrin Zimmermann, Matthias Nauck, Uwe Völker, Henry Völzke, Reiner Biffar, Christian Schulz, Julia Mayerle, Frank U. Weiss, Georg Homuth, Markus M. Lerch

**Affiliations:** 1 Department of Medicine A, University Medicine Greifswald, Greifswald, Germany; 2 Department of Functional Genomics, Interfaculty Institute for Genetics and Functional Genomics, University Medicine Greifswald, Greifswald, Germany; 3 Research Group Computational Systems Medicine, Chair of Experimental Bioinformatics, TUM School of Life Sciences Weihenstephan, Technical University of Munich, Freising-Weihenstephan, Germany; 4 Institute of Clinical Molecular Biology, Kiel University, Kiel, Germany; 5 Friedrich Loeffler Institute of Medical Microbiology, University Medicine Greifswald, Greifswald, Germany; 6 Institute of Clinical Chemistry and Laboratory Medicine, University Medicine Greifswald, Greifswald, Germany; 7 DZHK (German Centre for Cardiovascular Research), Partner Site Greifswald, University Medicine Greifswald, Greifswald, Germany; 8 Institute for Community Medicine, University Medicine Greifswald, Greifswald, Germany; 9 Department of Prosthetic Dentistry, Gerodontology and Biomaterials, University of Greifswald, Greifswald, Germany; 10 Department of Medicine II, University Hospital, LMU Munich, Munich, Germany

**Keywords:** Stomach diseases, Genetics research

## Abstract

*Helicobacter (H.) pylori* is the most important cause for peptic ulcer disease and a risk factor for gastric carcinoma. How colonization with *H. pylori* affects the intestinal microbiota composition in humans is unknown. We investigated the association of *H. pylori* infection with intestinal microbiota composition in the population-based cohort Study-of-Health-in-Pomerania (SHIP)-TREND. Anti-*H. pylori* serology and *H. pylori* stool antigen tests were used to determine the *H. pylori* infection status. The fecal microbiota composition of 212 *H. pylori* positive subjects and 212 matched negative control individuals was assessed using *16S rRNA* gene sequencing. *H. pylori* infection was found to be significantly associated with fecal microbiota alterations and a general increase in fecal microbial diversity. In infected individuals, the *H. pylori* stool antigen load determined a larger portion of the microbial variation than age or sex. The highest *H. pylori* stool antigen loads were associated with a putatively harmful microbiota composition. This study demonstrates profound alterations in human fecal microbiota of *H. pylori* infected individuals. While the increased microbiota diversity associated with *H. pylori* infection as well as changes in abundance of specific genera could be considered to be beneficial, others may be associated with adverse health effects, reflecting the complex relationship between *H. pylori* and its human host.

## Introduction

The Gram-negative proteobacterium *Helicobacter (H.) pylori* infects the gastric mucosa of approximately 50% of the global human adult population. It represents the major pathogen in the pathophysiology of diverse gastrointestinal conditions including gastritis, peptic ulcer disease, gastric adenocarcinoma, and mucosa-associated lymphoma^1^. In addition, several extra-gastrointestinal disorders such as iron deficiency anemia or idiopathic immunocytopenic thrombopenia have been associated with *H. pylori* infection^2^. However, despite chronic gastritis being histologically detectable in almost all cases^3^, the majority of affected individuals remain clinically asymptomatic during their lifetime.

In order to survive the hostile gastric environment, specifically gastric acid, *H. pylori* produces an alkaline ammonium cloud and moves towards the bicarbonate-rich mucous layer^4^. Although *H. pylori* does not invade the gastric epithelial layer its outer membrane proteins allow attachment to the epithelium^5^. Once infection is established, *H. pylori* persistently colonizes the gastric mucosa and dominates the gastric microbiome^6^. In Mongolian gerbils, *H. pylori* colonization has also been associated with changes in the large intestinal microbiota^7^. *H. pylori* infected transgenic insulin-gastrin mice were shown to have increased microbiota richness not only in the stomach, but also in cecal and colonic samples compared to non-infected controls^8^. However, no specific taxa with significantly altered abundance could be identified in their colon. In humans, only small studies analyzed changes in intestinal microbiota during and after eradication therapy of *H. pylori*^9–11^. Due to the dramatic effect of the antibiotics used for *H. pylori* eradication in those studies, changes in the microbiome cannot unambiguously be attributed to the absence of the pathogen. Older studies relied on culturing techniques^12^ which are inherently inappropriate to investigate the predominantly anaerobic gut milieu. In the present study we analyzed fecal microbiota profiles generated by *16S rRNA* gene sequencing of 212 *H. pylori* infected and 212 phenotypically matched control individuals from the population-based Study-of-Health-in-Pomerania-TREND (SHIP-TREND)^13^.

## Results

Phenotypic matching of the 212 *H. pylori* infected and 212 *H. pylori* negative subjects was performed to control for putative confounders known to influence intestinal microbiota such as age, sex, body mass index (BMI), alcohol consumption, smoking, proton pump inhibitor (PPI) intake, and diet^14–18^. A history of peptic ulcer disease was also considered for matching because affected individuals were more likely to have been subjected to eradication therapy which is assumed to influence gut microbiota^10^. After matching *H. pylori* cases and controls exhibited similar distribution patterns for all accounted phenotypic variables (Table 1 and Supplementary Table S1). None of the selected individuals were under antibiotic therapy at the time of sample collection.

**Table 1:** Phenotype variables of *H. pylori* infected cases and matched *H. pylori* negative controls.

	Controls (n = 212)	*H. pylori* cases (n = 212)	p-value
Age (years)	53.0 (43.0–63.0)	53.0 (44.0–62.0)	0.826
Male sex (%)	42.5	42.9	1
BMI (kg/m²)	27.3 (24.5–30.3)	27.3 (24.7–29.8)	0.493
Alcohol consumption (g/d)	4.1 (1.1–10.4)	3.9 (1.4–9.1)	0.851
Current smokers (%)	17.0	18.4	0.799
PPI users (%)	1.9	2.4	1
Individuals with history of PUD (%)	4.2	3.3	0.800

Continuous variables are expressed as median (1st–3rd quartile). Binary variables are given as percentages. The statistical significance was assessed using the Mann-Whitney test for continuous and the Fisher’s exact test for binary variables. BMI: Body mass index; PPI: Proton pump inhibitor; PUD: Peptic ulcer disease; n: number of individuals.

### Beta diversity analysis of *H. pylori* infected individuals as compared to non-infected controls

**Figure 1:**
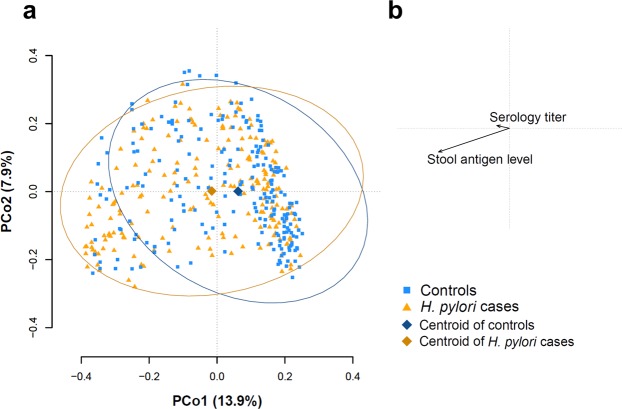
Principal coordinate analysis (PCoA) based on Bray-Curtis dissimilarity of *H. pylori* infected individuals and controls. (**a**) Shown are PCo1 and PCo2 of 424 gut microbiota samples. Orange triangles represent samples from *H. pylori* infected (n = 212) and blue squares samples from control individuals (n = 212), respectively. The centroids of both groups are displayed as diamonds and the respective samples are surrounded by a 95% data ellipse. *H. pylori* infected cases are shifted from controls. **(b)** Contribution of *H. pylori* stool antigen level and serology titer to beta-diversity. The association of *H. pylori* stool antigen level clearly exceeds that of serology titer.

Beta diversity analysis estimates how samples differ from each other. We used the commonly applied Bray-Curtis dissimilarity which is calculated based on the minimal shared abundance of each taxon. Thus, dual absence of taxa is not treated as similarity. Figure 1 shows the result of a principal coordinate analysis (PCoA) based on Bray-Curtis dissimilarity including all 424 microbiota samples. *H. pylori* infection was associated with a clear shift mainly along the first principal coordinate axis. Permutational analysis of variance (PERMANOVA) based on Bray-Curtis dissimilarity confirmed a significant shift of *H. pylori* cases compared to controls (r² = 0.011, p < 0.001). Association of continuous *H. pylori* antigen levels with beta-diversity explained even more variation (r² = 0.023, p < 0.001). The association with continuous *H. pylori* antibody levels was much weaker (r² = 0.006, p = 0.014). In a next step, we investigated whether *H. pylori* antibody or *H. pylori* stool antigen levels showed a significant association with beta-diversity only within the group of controls or *H. pylori* cases, respectively. No significant association was found within the group of controls. Within the group of *H. pylori* cases, *H. pylori* antibody levels did not show an association with beta-diversity. In contrast, continuous *H. pylori* antigen levels were associated with distinct changes in beta-diversity (r² = 0.029, p < 0.001). This effect size was even larger than that of age (r² = 0.015, p = 0.005) or sex (r² = 0.018, p = 0.002). No significant associations with beta-diversity were found for BMI, alcohol, smoking, use of PPI, or history of peptic ulcer disease in the group of *H. pylori* cases.

### Alpha diversity analysis of *H. pylori* infected individuals compared with non-infected controls

**Figure 2:**
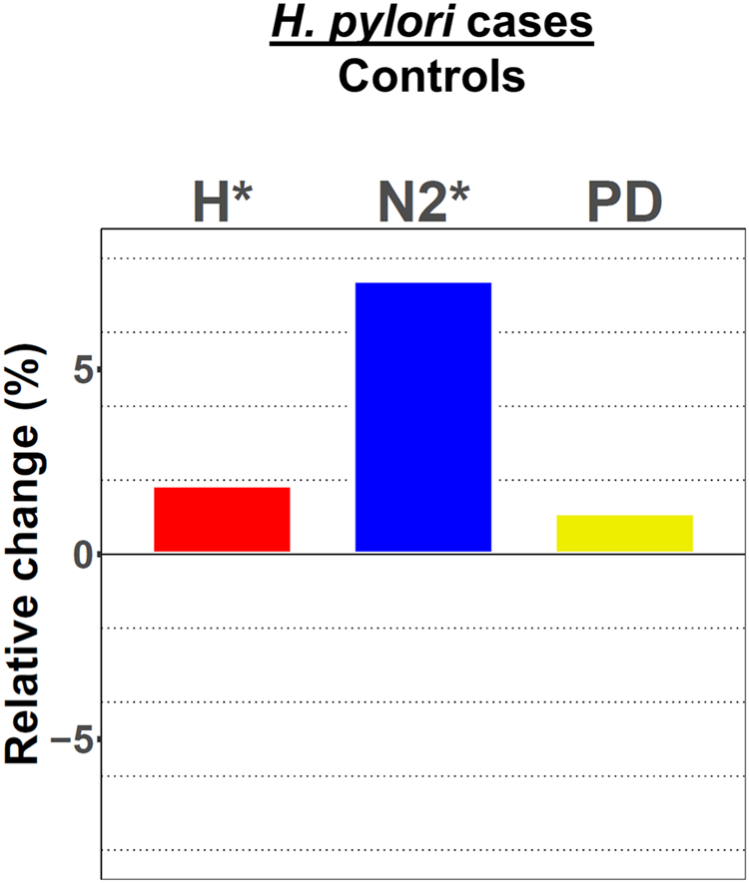
Alpha diversity analysis. Bars depict the relative change of the mean for Shannon diversity index (H, red), Simpson diversity number (N2, blue), and Phylogenetic diversity (PD, yellow) of *H. pylori* positive cases (n = 212) as compared to controls (n = 212). *Indicates a significant result (p < 0.05) according to t-test or Mann-Whitney test for H, N2, and PD, respectively.

Alpha diversity estimators characterize the diversity of an ecological community within a sample. We determined several alpha diversity indices with different emphases. The ‘Shannon diversity index’ (H) and the ‘Simpson diversity number’ (N2) include information about the number of different operational taxonomic units (OTUs), as well as the abundance of each taxon in the respective samples. The ‘Phylogenetic diversity’ tries to predict the genetic relatedness of the taxa in each sample. We compared the alpha diversity scores between *H. pylori* cases and controls using the parametric two-tailed t-test in case of the normally distributed ‘Shannon diversity index’ (Supplementary Fig. S1). In case of the non-normal distributed ‘Simpson diversity number’ and ‘Phylogenetic diversity’ (Supplementary Fig. S1) the two-tailed Mann-Whitney test was applied. Alpha diversity estimations revealed significantly higher scores in *H. pylori* cases compared to controls for ‘Shannon diversity index’ and ‘Simpson diversity number’ (Table 2 and Fig. 2). Although the median and mean ‘Phylogenetic diversity’ scores of *H. pylori* cases were also higher compared to controls, this was not significant.

**Table 2:** Alpha diversity estimations.

	Controls	*H. pylori* cases	p-value
Shannon diversity index (H)	4.03 (3.75–4.29) 4.01 ± 0.03	4.12 (3.87–4.31) 4.09 ± 0.02	0.032* (t-test)
Simpson diversity number (N2)	25.23 (19.36–32.79) 26.92 ± 0.76	26.73 (21.83–34.01) 28.91 ± 0.74	0.026* (MW)
Phylogenetic diversity (PD)	53.32 (44.07–64.70) 54.30 ± 0.97	55.84 (44.99–64.58) 54.91 ± 0.85	0.648 (MW)

Alpha diversity comparison between *H. pylori* negative controls (n = 212) and infected cases (n = 212). Scores are expressed as median (1st–3rd quartile) and mean ± SEM. The statistical significance was assessed using the t-test or Mann-Whitney test (MW) for normally or non-normally distributed scores, respectively. *Indicates a significant test result.

### Alterations of the intestinal microbiota in *H. pylori* infected individuals

In the complete study sample, the most abundant taxa at genus level were *Bacteroides*, unclassified members of the family *Ruminococcacae*, *Faecalibacterium*, and *Prevotella* comprising 41.4% of all microbiota. Supplementary Figs. S2 and S3 show the individual and the average composition of all participants at phylum and genus level, respectively.

**Figure 3:**
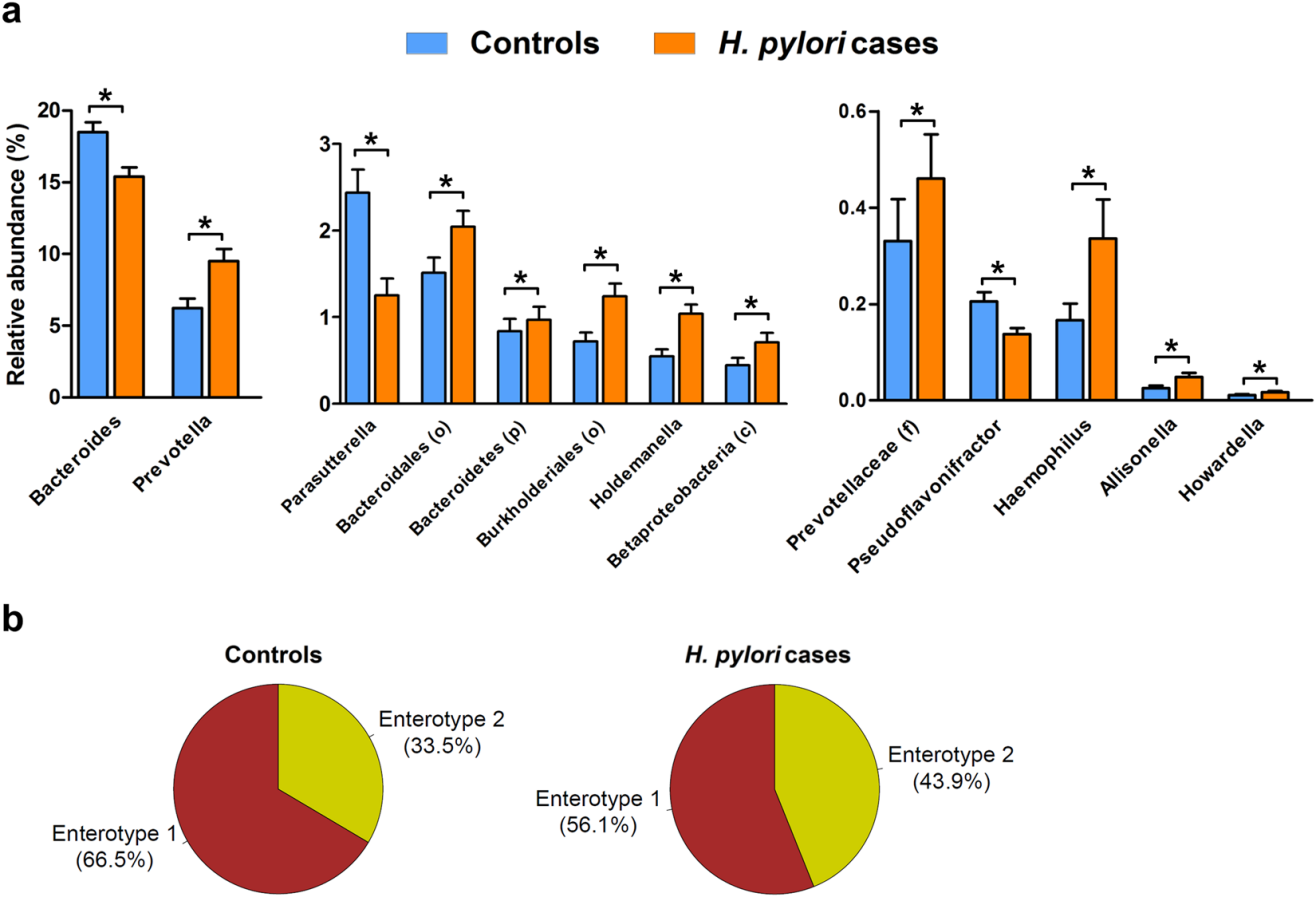
Alterations of intestinal microbiota in *H. pylori* infected individuals. (**a**) Barplots (mean + SEM) are showing all taxa at genus level with significantly different (q < 0.05, Mann-Whitney test) abundance between controls (blue, n = 212) and *H. pylori* infected (orange, n = 212) individuals. Lower case letters in brackets indicate taxonomic rank of unclassified taxa at genus level: class (c), family (f), order (o), or phylum (p). (**b**) Pie charts showing the distribution of enterotype 1 (*Bacteroides*-dominated, brown) and enterotype 2 (*Prevotella-*dominated, yellow) in controls (left) and *H. pylori* cases (right).

Comparing genus abundance data between *H. pylori* cases and controls, we identified 13 taxa, namely *Bacteroides*, *Prevotella*, *Parasutterella*, *unclassified Bacteroidales* (order), *unclassified Bacteroidetes* (phylum), *unclassified Burkholderiales* (order), *Holdemanella*, *unclassified Betaproteobacteria* (class), *unclassified Prevotellaceae* (family), *Pseudoflavonifractor*, *Haemophilus*, *Allisonella*, and *Howardella*, that were differentially abundant in *H. pylori* cases. These taxa represented 32.6% of the total microbial abundance in the complete cohort. The majority of identified taxa including the highly abundant *Prevotella* were associated positively with *H. pylori* infection (Fig. 3a and Supplementary Table S2), whereas the most abundant genus *Bacteroides* was reduced by 16.6% in *H. pylori* cases compared to controls.

*Arumugam et al*. described the existence of specific enterotypes that are either dominated by *Bacteroides* (enterotype 1), *Prevotella* (enterotype 2), or *Ruminococcus* (enterotype 3)^19^. To investigate whether the *H. pylori* carrier status affects the enterotype distribution, we performed enterotype clustering similar to the approach described by *Arumugam et al*. We found only two enterotypes in our dataset either dominated by *Bacteroides* or *Prevotella*. *Ruminoccous* or *unclassified Ruminococcaceae* were present in both groups and did not constitute a unique cluster. In controls, the enterotype 1 was present in 66.5% and enterotype 2 in 33.5% of cases. Analysis of *H. pylori* carriers revealed a shift from enterotype 1 (56.1%) towards enterotype 2 (43.9%) compared to controls (p = 0.036; Fisher’s exact test), (Fig. 3b).

### Association of intestinal microbiota with *H. pylori* stool antigen load in *H. pylori* infected individuals

**Figure 4:**
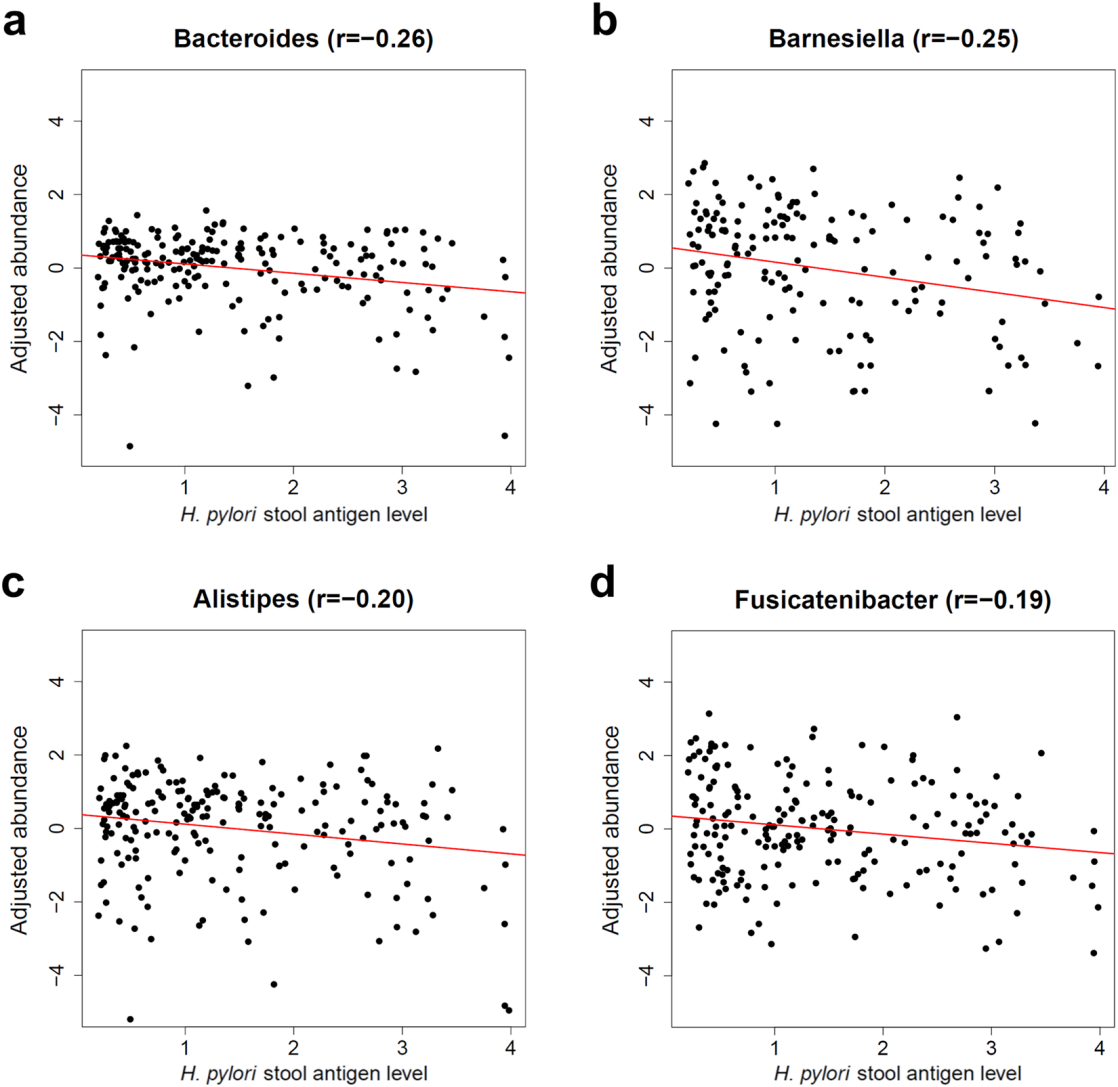
Genus association with *H. pylori* stool antigen load within *H. pylori* infected individuals. Shown are the age and sex adjusted abundance values (y-axis) of the four genera that were significantly associated with the *H. pylori* stool antigen level (x-axis) in *H. pylori* infected individuals. Only samples with presence of the respective genera were included. **(a)** *Bacteroides*, **(b)** *Barnesiella*, **(c)** *Alistipes*, and **(d)** *Fusicatenibacter*. r: Pearson correlation coefficient.

The results of the beta-diversity analysis revealed a significant microbiota variation even within the group of the 212 *H. pylori* cases with respect to the individual *H. pylori* load determined by stool antigen test. Hence, we performed linear regression analysis to identify the genera that are associated with the *H. pylori* stool antigen load. We analyzed all genera present in at least ten percent of all samples. Due to the reduced statistical power in this smaller data subset we focused on more prominent taxa by additionally excluding all genera with a mean abundance of less than or equal to 0.1% and all unclassified taxa at genus level. This analysis identified four genera that were all negatively associated with *H. pylori* stool antigen load, namely *Bacteroides* (q = 0.003), *Barnesiella* (q = 0.018), *Alistipes* (q = 0.035), and *Fusicatenibacter* (q = 0.046), (Fig. 4 and Supplementary Table S3). Performing a similar analysis for the individual *H. pylori* serology level instead of the stool antigen load did not yield a significant association.

## Discussion

We investigated changes in the intestinal microbial community structure of *H. pylori* infected individuals, finding significant alterations of the microbiota composition and diversity. Strikingly, in *H. pylori* cases, the alpha diversity estimators ‘Shannon diversity index’ and the ‘Simpson diversity number’ exhibited generally higher scores, indicating increased microbial diversity. High diversity is generally considered to be an indicator of a healthy gut microbiome, while a decrease is associated with poorer health or unhealthy habits. For example, a plant rich diet increases alpha diversity^20^, whereas a Western lifestyle or obesity are associated with reduced bacterial diversity^21,22^. In addition, conditions such as recurrent antibiotic-associated diarrhea^23^, Crohn’s disease^24^, or ulcerative colitis^25^, have all been reported to be associated with reduced intestinal diversity. Considering these findings, the observed positive correlation of *H. pylori* infection with increased diversity suggests a putative beneficial effect of *H. pylori* as it may strengthen the host’s resilience against microbiome perturbations or gastrointestinal infections. In more detail, a total of 13 taxa exhibited different abundance values between *H. pylori* infected cases and controls. We found *Parasutterella* to be decreased in case of *H. pylori* infection, a genus that has been reported to be increased in the ileal submucosa of Crohn’s disease patients^26^. *H. pylori* infected individuals exhibited increased levels of the facultative pathogen *Haemophilus* and decreased levels of *Pseudoflavonifractor*. The latter has been reported to be involved in the production of short-chain fatty acids (SCFA) such as butyrate^27^. SCFA represent an important energy source for colonic epithelia^28^ and have been shown to modulate the immune response by regulating colonic regulatory T-cell function and alleviate experimental colitis in rodents^29^. Thus, the decrease of the SCFA producer *Pseudoflavonifractor* may be disadvantageous for the human host as well.

In addition to the binary comparison of *H. pylori* infected individuals with controls we addressed putative differences within the group of *H. pylori* positive individuals and found that the *H. pylori* antigen load was associated with larger alterations in the fecal microbiota than age or sex. Of note, this association was not found for variations in the *H. pylori* antibody titer. The *H. pylori* antigen load was negatively associated with the four genera *Bacteroides*, *Barnesiella*, *Fusicatenibacter*, and *Alistipes*. Several of these taxa have previously been attributed health promoting features. The presence of *Barnesiella* was reported to be positively associated with higher eradication rates of antibiotic-resistant bacteria after fecal microbiota transplantation in mice^30^ and humans^31^, whereas higher rates of chemotherapy-related blood stream infections were observed in individuals with reduced *Barnesiella* counts^32^. *Fusicatenibacter* is known to be involved in SCFA production and to produce lactic acid^33^. It may therefore exert anti-inflammatory properties as shown for other lactic acid producing bacteria^34^. Finally, *Alistipes* is a supposed producer of the SCFA butyrate, which can alleviate intestinal inflammation^35^. Consequently, the observed alterations in individuals with a particularly high *H. pylori* antigen load may together cause adverse consequences for the human host. The putative health benefits mediated by the increased microbial diversity associated with *H. pylori* infection could be counteracted when *H. pylori* load is high.

*Arumugam et al*. first reported three different fecal microbiome clusters they designated as enterotypes^19^. It was proposed that each enterotype is characterized by a marked occurrence of *Bacteroides* (enterotype-1), *Prevotella* (enterotype-2), or *Ruminococcus* (enterotype-3). These authors also reported that enterotype-1 generates energy primarily by fermentation of carbohydrates and proteins, since genes encoding for galactosidases, hexosaminidases and proteases were more common. In contrast, the dominating genus of enterotype-2, *Prevotella*, is supposed to be a mucin degrader^36^. Furthermore, *Arumugam et al*. did not find a significant correlation of any of these enterotypes with BMI, sex, or age. In a later study, only two clusters dominated by *Bacteroides* or *Prevotella* were confirmed^37^. *Ruminococcus* which did not form an individual cluster was subsequently fused with that of *Bacteroides*. Both remaining clusters were primarily separated by dietary effects. The *Bacteroides* dominated enterotype was associated with the intake of animal protein and saturated fats, whereas the *Prevotella* dominated enterotype was associated with the intake of carbohydrates and a plant-based diet. In the present study, using a similar approach to that of *Arumugam et al*., two clusters either dominated by *Bacteroides* or *Prevotella* could be identified. The *Prevotella* dominated enterotype 2 was more common in *H. pylori* infected individuals. As our dataset was matched with respect to diet, this observation could not merely be explained by differences in intake of animal proteins or plant based products. There is currently an ongoing debate about the general concept of enterotypes. It was proposed that the clustering results which revealed separated enterotypes were rather guided by the dominant abundance of *Bacteroides* or *Prevotella*, respectively, and did not result from the presence of consistent microbial communities in each cluster^38^. Furthermore, it was emphasized that *Bacteroides* and *Prevotella* may form continuous gradients rather than being clearly segregated into two groups^39,40^. While contributing to the debate about the adequacy of the term ‘enterotype’ is beyond the scope of this study, we found *H. pylori* infection to be associated with a shift of the intestinal microbiota towards a *Prevotella* dominated microbiome, irrespective of the ‘enterotypes’ concept.

The underlying cause of the observed fecal microbiota changes associated with *H. pylori* infection is unknown. The altered gut microbiome in *H. pylori*-infected Mongolian gerbils has been explained by gastric hypochlorhydria^7^. It seems, indeed, plausible that a reduced production of gastric acid would promote the passage of acid-sensitive bacteria leading to an enriched diversity of the intestinal microbiome. During *H. pylori* infection there are generally two periods characterized by reduced gastric acid secretion: First, the initial infection phase can be followed by acute gastritis with temporarily impaired production of gastric acid^4^. Second, in a later stage of *H. pylori* infection many individuals develop pangastric, hypoacidic chronic gastritis caused by destruction of parietal cells^41^. However, this simple model would not be supported by the observation that gastric acid-suppressed PPI users were found to exhibit a reduced intestinal diversity^14,16^. Modulation of the distal gut microbiota diversity by *H. pylori* is therefore more complex. It has been proposed that the impact of *H. pylori* infection is not restricted to the gastrointestinal tract. In murine models, *H. pylori* demonstrated immunoregulatory features by preventing allergic asthma through the induction of regulatory T cells via IL-18 mediated tolerogenic reprogramming of dendritic cells, which then ensures the persistence of the pathogen^42,43^. In humans *H. pylori* could also regulate the intestinal microbial community composition in a similar fashion by modifying the host’s immune response.

In the complete dataset including *H. pylori* cases and controls, we did not find any stool microbiota profile with presence of the genus *Helicobacter*. Although PCR based approaches have managed to detect *H. pylori* in fecal samples before, these used distinct sample preparation (e. g. immunomagnetic separation) and/or specific primers for *H. pylori* genes for its detection^44^. Yet, other investigations failed to detect fecal *H. pylori* DNA even when using specific primers^45^. As the gastric pathogen *H. pylori* may mostly not survive under the anaerobic conditions of the distal intestine its fecal DNA concentrations are likely very low compared to the majority of anaerobic bacteria. It may therefore escape detection in fecal samples when investigating with an untargeted approach such as *16S rRNA* gene sequencing.

The strength of the present study includes the large study population, the high specificity of the *H. pylori* diagnosis based on the assessment of both serology and stool antigen testing, and the thorough phenotypic matching of factors known to influence intestinal microbiota in order to avoid bias. Its main limitations result from the design as an association study. It may be possible that the *H. pylori* stool antigen levels were influenced by the gut microbiota composition, rather than the *H. pylori* load determining the gut microbiota. However, given the high sensitivity and specificity of the *H. pylori* stool antigen test in comparison to histology, culture, rapid urease test, or urea breath test^46^, it seems unlikely that the antigen levels were affected by gut microbiota to a great extent. A poorer performance of the stool antigen test could be predicted if defined gut bacteria would specifically degrade the target antigen.

It has been assumed that humans have been infected with *H. pylori* for at least ~100,000 years^47^ and distinctly more than half of the population in many developing and emerging countries are currently infected. In light of the sometimes life-threatening disorders that can arise in *H. pylori* infected individuals, this long lasting evolutionary relationship may be explainable by counterbalancing beneficial effects of the infection. Among them are the already mentioned protection against atopic diseases described in murine models^42^ and, according to our data, possible suppression of other gastrointestinal pathogens by increasing microbial diversity. Therefore, *H. pylori* eradication may trigger unwanted detrimental gut microbiota alterations, namely a reduced microbiota diversity, in individuals with low-grade infection. This underlines the need for careful decision making regarding *H. pylori* eradication in each individual. However, the complex relationship between *H. pylori* and its human host is once again demonstrated by the probably adverse microbial alterations found in individuals with high *H. pylori* antigen load. The large proportion of *H. pylori* infected individuals in the global population emphasizes the importance of this finding. As the applied method of *16S rRNA* gene sequencing only allows taxon identification, future whole-genome sequencing approaches will have to define the functional potential of the enriched taxa in *H. pylori* cases.

## Methods

### Study participants

The longitudinal population-based Study-of-Health-in-Pomerania (SHIP) aims to determine the incidence and prevalence of common risk factors, subclinical disorders and clinical disease^13^. It comprises the two independent cohorts SHIP (recruitment 1997–2001) and SHIP-TREND (recruitment 2008–2012) with re-evaluations in 5-year intervals. All participants provided written informed consent. The study was approved by the ethics committee of the University Medicine Greifswald and carried out in accordance with its regulations.

### *H. pylori* stool antigen test and serology

The *H. pylori* stool antigen test was performed using the *H. pylori* antigen ELISA Kit (Immundiagnostik AG, Bensheim, Germany). According to the manufacturer’s instructions, 100 mg stool were used per sample and all samples with an OD greater than or equal to 0.015 at 450 nm were considered positive for *H. pylori*. The *H. pylori* serology status was evaluated by anti-*H. pylori* serum IgG determination using the enzyme-linked immunoassay Pyloriset EIA-G III (Orion Diagnostica, Espoo, Finland). A positive test result was considered for antibody titers equal or greater to 20 U/ml^48^.

### Definition of *H. pylori* infection

The initial dataset comprised 931 SHIP-TREND participants with available *H. pylori* stool antigen test as well as serology results. *H. pylori* infection was assumed when both, *H. pylori* stool antigen test and *H. pylori* serology, were positive. *H. pylori* negativity was assigned when both *H. pylori* stool antigen test and serology were negative. According to these criteria, 228 individuals were positive (24.5%) and 500 negative (53.7%) for *H. pylori* infection. A total of 183 (19.7%) participants demonstrated exclusively a positive *H. pylori* serology and 20 (2.1%) participants exclusively a positive stool antigen test. Of the total dataset of 931 individuals, we excluded 12 participants due to missing phenotype data and further 6 individuals because of an intake of antibiotics at the time of sample collection. This resulted in 221 *H. pylori* infected individuals of whom stool DNA for microbiota analysis by *16S rRNA* sequencing was available for 212 individuals. As controls, 212 samples from individuals with both negative *H. pylori* stool antigen test as well as *H. pylori* serology were selected. All control samples were matched with respect to age, sex, BMI, alcohol consumption, smoking, PPI usage, history of peptic ulcer disease, and dietary habits using the ‘R’^49^ package ‘MatchIt’ (option ‘nearest’)^50^.

### *16S rRNA* gene sequencing

Sequencing was performed as described before^51^. In brief, isolated DNA from fecal samples was used for amplification of the V1-V2 region of bacterial *16S rRNA* genes on a MiSeq platform (Illumina, San Diego, USA). MiSeq Fast-Q files were created by CASAVA 1.8.2 (https://support.illumina.com/sequencing/sequencing_software/casava). After quality trimming of sequences with Sickle (https://github.com/najoshi/sickle), forward and reverse reads were merged and filtered using VSEARCH^52^. Subsequently all reads were quality filtered by FastX Toolkit (http://hannonlab.cshl.edu/fastx_toolkit). At this step, only reads with a quality score of at least 30 (error probability 1 in 1,000) per base in 95% of sequenced nucleotides were included. To reduce redundancy among sequences de-replication was performed. OTUs were clustered using VSEARCH demanding a minimum sequence similarity of 97%. After chimera filtering by USEARCH^53^ each sample was normalized to 10,000 reads by random selection. Four of the 424 samples contained slightly less than 10,000 reads (9897, 9864, 9815, and 9408 reads). For assignment of taxonomy the SINTAX classifier was used^54^. A confidence of at least 80% for each taxonomic rank was ascertained. All taxa with a confidence below 80% were assigned to an arbitrary taxon as unclassified family, order, class, phylum, or bacteria, respectively.

### Data analysis

All statistical analyses were performed using ‘R’. Bar plots were created with GraphPad Prism 5 (GraphPad Software, San Diego, USA). For calculation of ‘Shannon diversity index’ and ‘Simpson diversity number’ the R package ‘vegan’^55^ was used based on OTU counts. ‘Phylogenetic diversity’ was determined using the package ‘picante’^56^. Q-Q plots for normality assessment were generated using the *qqplot* function of the ‘stats’ package. The Bray-Curtis dissimilarity was calculated based on genus level data using the ‘vegan’ function *vegdist*. PCoA was performed with the *cmdscale* function from the ‘vegan’ package. Square root transformation of the Bray-Curtis dissimilarity was performed prior to the ordination to avoid negative eigenvalues. To determine the contribution of phenotypic variables to the Bray-Curtis dissimilarity, PERMANOVA was done using the ‘vegan’ function *adonis* and the significance assessed by 10,000 permutations. For regression of phenotypic variables on the two major principal coordinate axes the ‘vegan’ function *envfit* was used. The two-tailed Mann-Whitney test was performed for assessment of significance in case of continuous data. A two-tailed t-test was applied for significance assessment of the normally distributed ‘Shannon diversity index’. Fisher’s exact test was utilized for categorical data. Comparison of the relative abundance of all taxa that were present in at least ten percent of all samples at genus level between *H. pylori* cases and control individuals was performed using the two-tailed Mann-Whitney test. The resulting p-values were adjusted for multiple testing following the Benjamini-Hochberg procedure and called ‘q-values’. Association of the *H. pylori* antigen load with individual genera within the group of *H. pylori* infected individuals was examined as follows: All classified genera present in at least ten percent of all samples and with a mean abundance of greater than 0.1% were analyzed using a linear regression model (‘R’ function *lm*) based on log transformed abundance data. For this analysis zero-values were ignored, i.e. treated as missing values, to avoid biased linear regression estimates due to inflation of zeros. The putative confounder age and sex were included in the model and resulting p-values adjusted for multiple testing following the Benjamini-Hochberg procedure. P-values or q-values < 0.05 were considered significant. All p- and q- values were rounded to three significant digits.

## Supplementary information

Supplementary tables.

Supplementary figures.

## Data Availability

All microbiome and phenotype data were obtained from the Study-of-Health-in-Pomerania (SHIP/SHIP-TREND) data management unit and can be applied for online through a data access application form (https://www.fvcm.med.uni-greifswald.de/dd_service/data_use_intro.php).
