# Supplementary material for: Helicobacter pylori infection associates with fecal microbiota composition and diversity
Source: Sci Rep. 2019 Dec 27;9:20100. doi: 10.1038/s41598-019-56631-4 (PMC6934578; doi:10.1038/s41598-019-56631-4)
Supplement: Supplementary file 2 — Supplementary figures. [file 41598_2019_56631_MOESM2_ESM.docx]

***Helicobacter pylori* infection associates with fecal microbiota composition and diversity**

Fabian Frost^1^; Tim Kacprowski^2,3^; Malte Rühlemann^4^; Corinna Bang^4^; Andre Franke^4^; Kathrin Zimmermann^5^; Matthias Nauck^6,7^; Uwe Völker^2^; Henry Völzke^8^; Reiner Biffar^9^; Christian Schulz^10^; Julia Mayerle^1,10^; Frank U Weiss^1^; Georg Homuth^#2^ & Markus M Lerch^#^*^1^

# Equally contributing senior authors

**Authors institutions:**

1) Department of Medicine A, University Medicine Greifswald, Greifswald, Germany

2) Department of Functional Genomics, Interfaculty Institute for Genetics and Functional Genomics, University Medicine Greifswald, Greifswald, Germany

3) Research Group Computational Systems Medicine, Chair of Experimental Bioinformatics, TUM School of Life Sciences Weihenstephan, Technical University of Munich, Freising-Weihenstephan, Germany

4) Institute of Clinical Molecular Biology, Kiel University, Kiel, Germany

5) Friedrich Loeffler Institute of Medical Microbiology, University Medicine Greifswald, Greifswald, Germany

6) Institute of Clinical Chemistry and Laboratory Medicine, University Medicine Greifswald, Greifswald, Germany

7) DZHK (German Centre for Cardiovascular Research), Partner Site Greifswald, University Medicine Greifswald, Greifswald, Germany

8) Institute for Community Medicine, University Medicine Greifswald, Greifswald, Germany

9) Department of Prosthetic Dentistry, Gerodontology and Biomaterials, University of Greifswald, Greifswald, Germany

10) Department of Medicine II, University Hospital, LMU Munich, Munich, Germany

**Supplementary Figures**


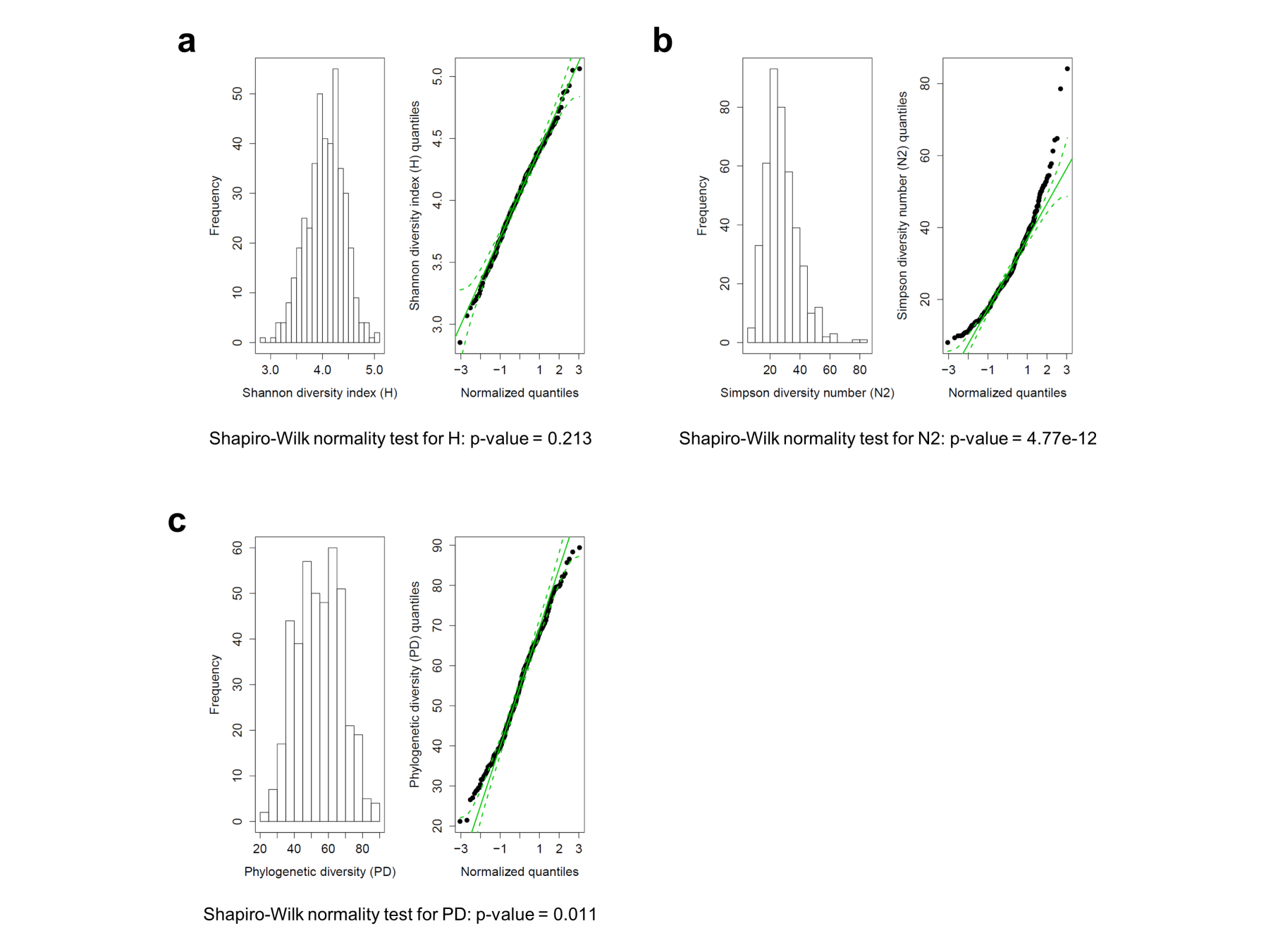


**Supplementary Figure S1: Data distribution of different alpha diversity indices.** Shown are histograms and corresponding Q-Q plots of **(a)** Shannon diversity index (H), **(b)** Simpson diversity number (N2), and **(c)** Phylogenetic diversity (PD). Data includes the complete study group (n=424). The diagonal green line depicts the reference line. The dashed green line denotes the 95%-confidence interval of the theoretical distribution. N2 and PD do not follow a normal distribution confirmed by a significant (p<0.05) Shapiro-Wilk normality test.

**
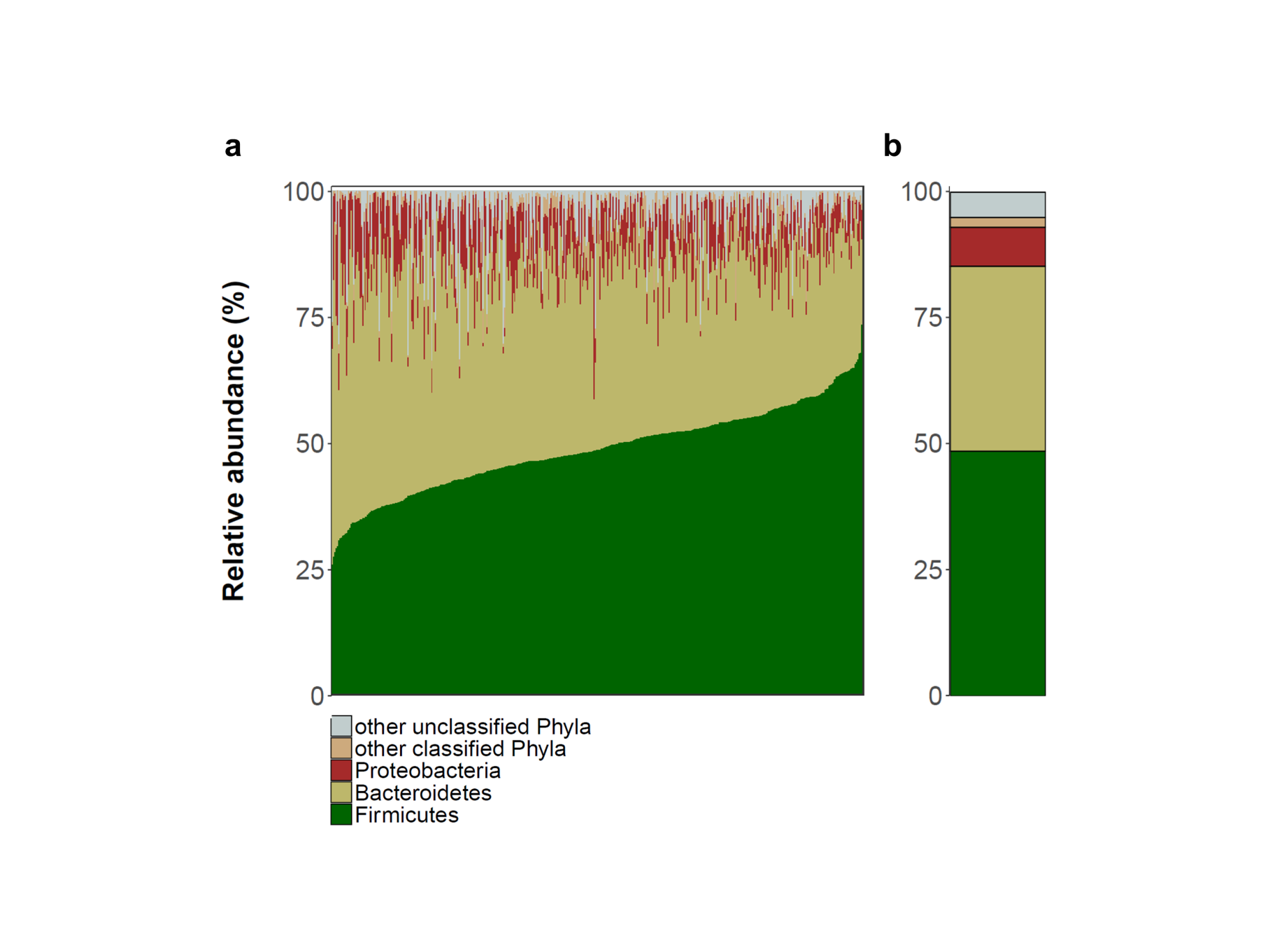
**

**Supplementary Figure S2: Microbiota composition at phylum level. (a)** Shown are stacked bar plots displaying the taxon distribution at phylum level for each sample (n=424). All samples are ordered by the relative abundance of the phylum *Firmicutes*. **(b)** Stacked bar plot shows the mean relative abundances at phylum level for the whole study group.


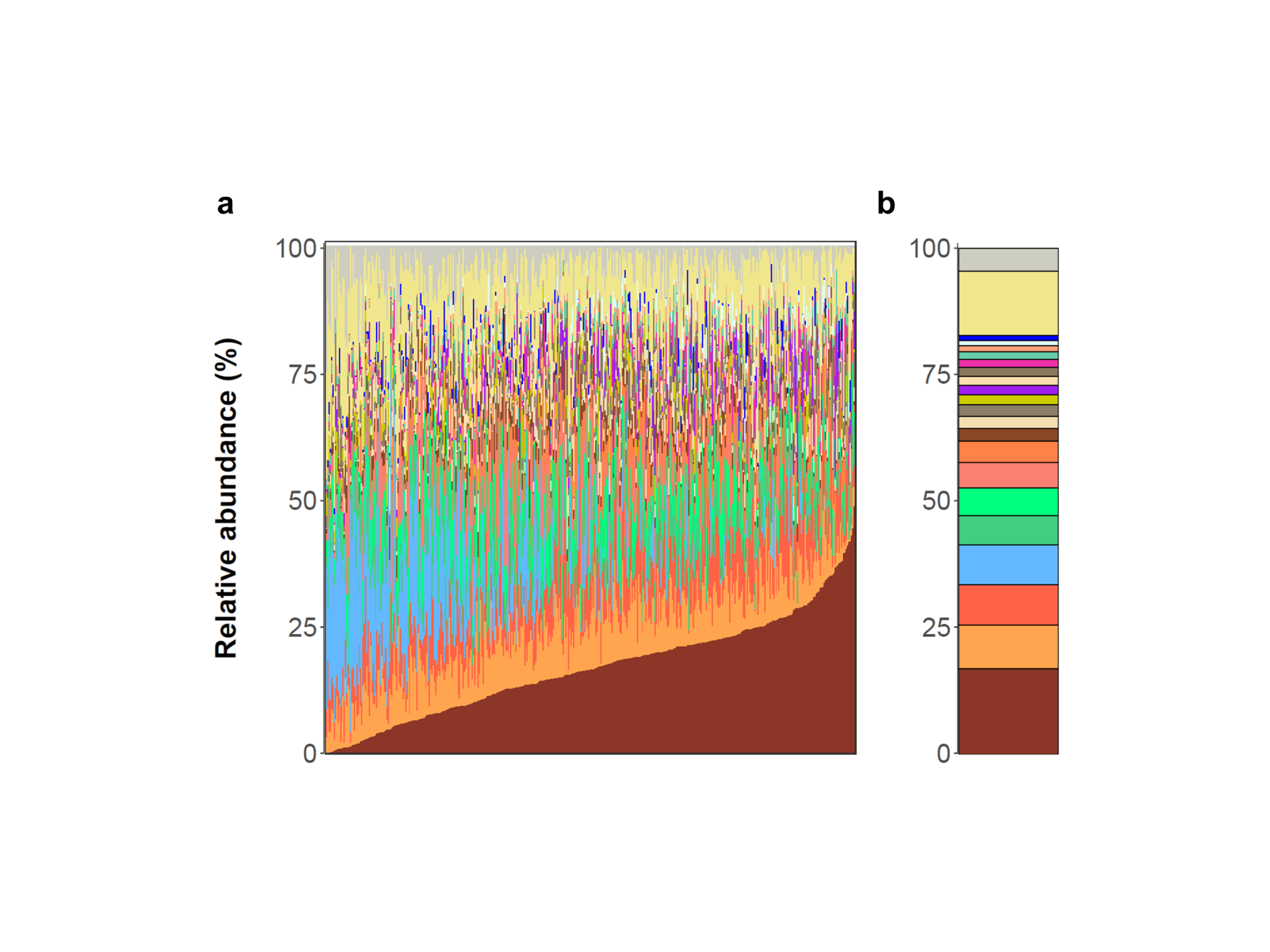


**
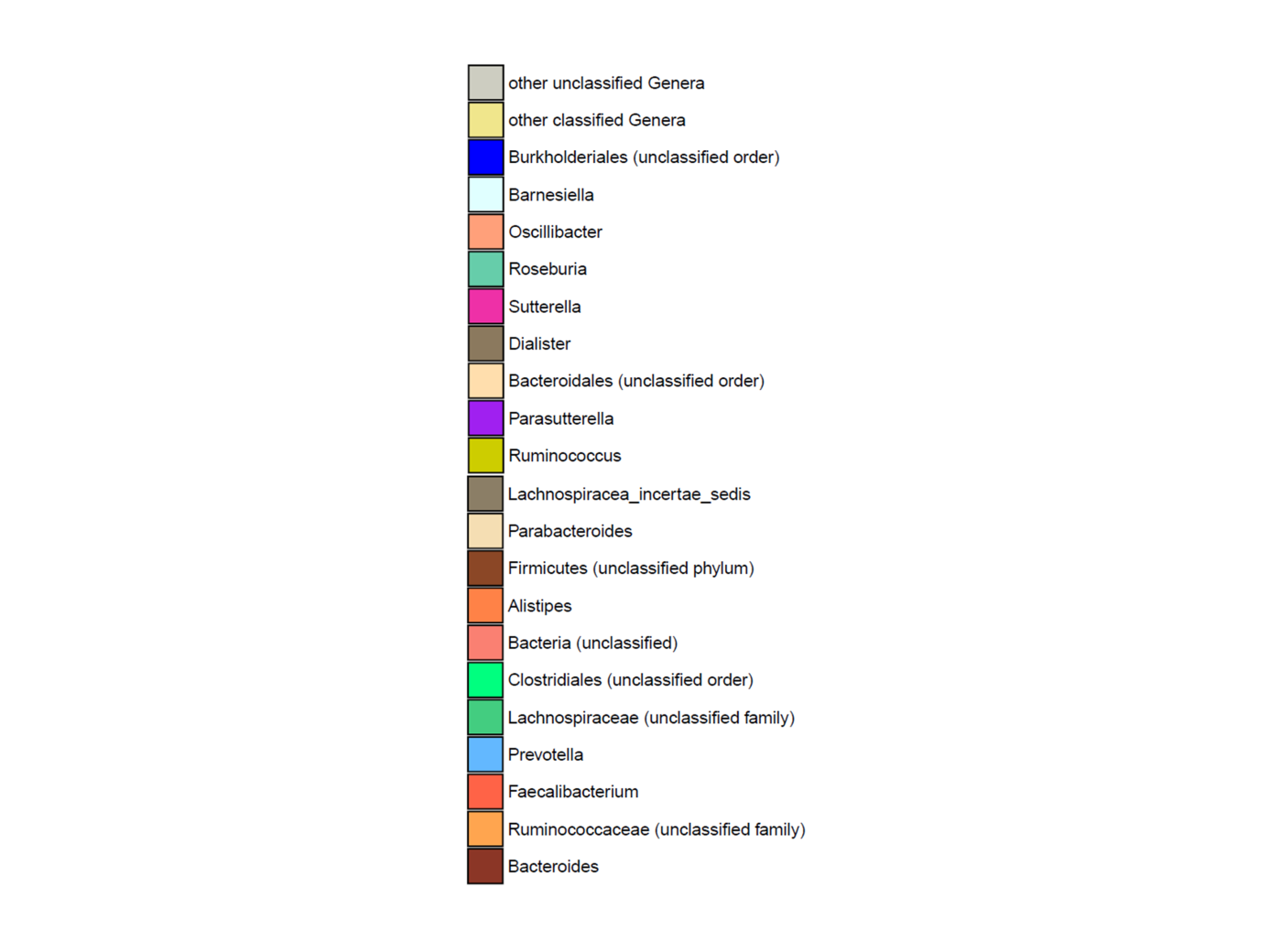
**

**Supplementary Figure S3: Microbiota composition at genus level. (a)** Shown are stacked bar plots displaying the taxon distribution at genus level for each sample (n=424). All samples are ordered by the relative abundance of the genus *Bacteroides*. **(b)** Stacked bar plot shows the mean relative abundances at genus level for the whole study group.
